# Evaluation of Genetic Parameters and Comparison of Stress Tolerance Traits in Different Strains of *Litopenaeus vannamei*

**DOI:** 10.3390/ani14040600

**Published:** 2024-02-12

**Authors:** Miao Shi, Song Jiang, Jianzhi Shi, Qibin Yang, Jianhua Huang, Yundong Li, Lishi Yang, Falin Zhou

**Affiliations:** 1 Key Laboratory of Efficient Utilization and Processing of Marine Fishery Resources of Hainan Province, Sanya Tropical Fisheries Research Institute, Sanya 572018, China; 2 Shenzhen Base of South China Sea Fisheries Research Institute, Chinese Academy of Fishery Sciences, Shenzhen 518108, China; 3 South China Sea Fisheries Research Institute, Chinese Academy of Fishery Sciences, Guangzhou 510300, China

**Keywords:** *Litopenaeus vannamei*, ammonia-N, pH, salinity, tolerance, heritability, genetic parameter

## Abstract

**Simple Summary:**

The *Litopenaeus vannamei* shrimp is of paramount economic importance and has risen to prominence as a leading species in global aquaculture. Within its natural settings, *Litopenaeus vannamei* is subjected to a variety of environmental stressors that could negatively impact its survival, growth, and reproductive success. This study was designed to create specific lineages of *Litopenaeus vannamei* populations for resilience evaluation under stress conditions such as ammonia-N, pH, and salinity. Utilizing an exhaustive animal model that incorporates both genders, we performed a genetic parameter analysis to assess the heritability of stress tolerance traits in *Litopenaeus vannamei*. Concurrently, correlations among these traits were examined. Our findings lay solid theoretical groundwork for the selective breeding of *Litopenaeus vannamei*, providing valuable insights into enhancing varieties and improving economic yields. Furthermore, this research effort plays a crucial role in advancing the health and sustainable development of the aquaculture industry.

**Abstract:**

*Litopenaeus vannamei* stands out globally in aquaculture for its fast growth, broad salt tolerance, disease resistance, and high protein levels. Selective breeding requires the precise estimation of the variance components and genetic parameters for important traits. This study formed lineages from 20 full sibling families of *L. vannamei*, with progenitors from Thailand and the USA. We then assessed the genetic resilience traits of juvenile shrimp from these families to high ammonia-N, high pH, and low salinity by performing a 96 h acute toxicity test. Mortality rates for the families under 96 h exposure to high ammonia-N, high pH, and low salinity were 19.52–92.22%, 23.33–92.22%, and 19.33–80.00%, respectively, showing significant variance in stress tolerance among families (*p* < 0.05). Survival heritability estimates, using threshold male and female models, were 0.44 ± 0.12 in high ammonia-N, 0.41 ± 0.12 in high pH, and 0.27 ± 0.08 in low salinity, respectively. Genetic correlations between growth and stress resistance traits varied from 0.0137 ± 0.2406 to 0.8327 ± 0.0781, and phenotypic correlations ranged from 0.0019 ± 0.0590 to 0.6959 ± 0.0107, indicating a low-to-high positive correlation significant at (*p* < 0.05). It was found that the survival rate of families No. 2 and No. 9 was higher under high ammonia-N and high pH stresses, while the survival rate of family No. 10 was higher under low salinity stress after comparing two selection criteria, the breeding values and phenotypic values. Thus, these three families are identified as potential breeding program candidates. Through the creation of a genetic parameter estimation model, the genetic variances across mating combinations for stress resistance traits were obtained and families with heightened stress resistance were identified, laying the groundwork for enhanced genetic selection of *L. vannamei*.

## 1. Introduction

*Litopenaeus vannamei* ranks among the top three shrimp species globally, representing 52.9% of total crustacean culture yield [1]. The combination of industrial high-density mariculture and increasing extreme weather events induces stress and diminishing growth and survival rates [2,3,4,5]. Consequently, genetic improvement in *L. vannamei*, aimed at enhancing stress resistance and adaptation to evolving aquaculture conditions, climate changes, and diverse farming practices, is critically important. Conducting stress tolerance tests for high ammonia-N, high pH, and low salt in *L. vannamei* is essential to precisely evaluate genetic parameters of stress tolerance traits. Moreover, comparing stress tolerance across family lines and performing correlation analysis are fundamental for the sustainable culture of *L. vannamei* [6].

Genetic parameters and breeding values are essential metrics used to assess genetic traits. The estimation results play a pivotal role in providing a comprehensive understanding and prediction of genetic trait performance across different genotypes for breeders. Such insights allow breeders to develop targeted breeding strategies based on expected results. By integrating these findings, breeders are empowered to make well-informed choices aimed at enhancing the breeding process overall. In assessing genetic parameters of resilience traits in aquatic species, Zhou [7] identified that the heritability of ammonia-N tolerance and low salt tolerance in *Penaeus monodon* were 0.11 ± 0.04 and 0.29 ± 0.08, respectively, with a moderate positive correlation observed between the breeding values for ammonia-N tolerance and freshwater stress tolerance. Similarly, He [8] observed significant variation in ammonia-N tolerance and high pH tolerance among *Fenneropenaeus chinensis* larvae from different lineages during an acute tapping assay, indicating a high selection potential for these traits. Wang [9] constructed 15 half-sibling families of *Larimichthys crocea*, estimating the genetic parameters for traits like low salt, low dissolved oxygen, and low pH tolerance, revealing heritabilities of 0.23 for both low dissolved oxygen and low pH tolerance, and a heritability of 0.10 for low salt tolerance. Jiang [10] estimated the genetic parameters for growth and low salt tolerance in *Haliotis diversicolor supertexta*, finding a heritability of 0.06 ± 0.02 for the latter trait. Furthermore, Wang [11] evaluated genetic parameters for growth and combined stress tolerance in *L. vannamei*, showing that these traits possess high and intermediate levels of heritability, respectively, suggesting that improvements in growth traits could indirectly benefit combined stress tolerance. The influence of genetic and environmental factors on phenotypic traits [12] underscores the importance of breeding new *L. vannamei* varieties with enhanced stress tolerance for the health and sustainable development of the shrimp industry.

In this study, 20 self-breeding and hybrid families of *L. vannamei* were developed by sourcing high-quality germplasm resources from Thailand and the United States. These families underwent ammonia-N, pH, and salinity stress tests over a 96 h period to assess their stress tolerance capabilities. The genetic analysis of survival characteristics in juvenile *L. vannamei* utilized threshold models for both males and females. The restricted maximum likelihood method (REML) was employed to evaluate genetic parameters related to carapace length, body length, body weight, and stress tolerance traits in *L. vannamei* [13,14]. The aim was to enhance the precision of breeding selection for *L. vannamei* by identifying families with higher tolerance levels. This endeavor sought to provide theoretical insights and valuable data to support the breeding science of *L. vannamei*.

## 2. Materials and Methods

### 2.1. Parental Material and Lineage Construction

The study was conducted at Hainan Lutai Marine Biotechnology Co., Ltd., employing two imported *L. vannamei* populations as parental shrimp: the Dingfeng strain (T) from Thailand and the Daynight Express strain (M) from the United States (Table 1). Prior to transferring the parental shrimp to the breeding pond, 50 individuals were randomly selected for sampling, with the first pleopods chosen as the primary site for screening common shrimp viruses such as WSSV, TSV and IHHNV, utilizing PCR technology. The outcomes were uniformly negative, with the screening aiming to prevent the vertical transmission of these viruses and guarantee the health of the subsequent generation of juvenile shrimp for the experiment. Following the virus screening and growth trait assessment, the parental shrimp were relocated to a holding pool for a brief acclimation period. After seven days of acclimation, the female shrimp underwent unilateral eyestalk ablation using a forceps ironing technique to promote gonadal maturation. Throughout this period, the water temperature was consistently maintained at 28 ± 0.5 °C, supplemented with 24 h oxygenation through nanotubes. Live sandworms and frozen squid were fed to the shrimp daily to fortify and ripen the gonads of the parent shrimp. Meanwhile, the hose siphon method was used for suction and sewage disposal. The families were constructed by double-row natural mating, and the gonadal maturity of the shrimp was checked at 17:00 every day. The gonadally mature females were individually transferred to 500 L spawning barrels, identified with an eyestalk ring. After overnight spawning, the shrimp were returned to their ponds at dawn, and any dead or unfertilized eggs were promptly removed within 24 h. Larval rearing was conducted individually for each spawning barrel, with offspring from each female designated as a distinct lineage, meticulously documented throughout the process. Within nine days, four self-breeding and hybrid populations were successfully established: T♀ × T♂ (TT), M♀ × M♂ (MM), T♀ × M♂ (TM), and M♀ × T♂ (MT), totalling 20 families.

### 2.2. Intermediate Breeding of Family Lines

From each family, 2000 larvae were randomly selected. Throughout the incubation phase, larvae exhibiting poor vitality and weak physical conditions were systematically removed, and the development of the families was tracked up to the juvenile stage. Each family retained 1000 juveniles, and upon reaching the postlarval stage 15 (PL_15_), 500 nauplii were randomly chosen and transferred to the cement pool in the roughing workshop to boost the young shrimp’s adaptability to aquatic environments. During the intermediate rearing phase, conditions such as salinity, temperature, larval density, feed type, water exchange, and aeration were uniformly maintained across all stages to reduce the impact of environmental variance on growth and development. The larvae that remained post-selection were placed into an indoor cement pool, and mixed as preparatory material for upcoming resilience tests concerning ammonia-N, pH, and salinity levels. When the shrimp attained a length of 4–5 cm, individuals of comparable size were randomly selected from each family for testing, with three replicates per group and 50 shrimp per replicate. The test shrimp displayed the following characteristics: TT body length (51.10 ± 6.50 mm), body weight (1.34 ± 0.45 g/tail); MM body length (49.90 ± 6.00 mm), body weight (1.30 ± 0.44 g/tail); TM body length (44.70 ± 5.00 mm), body weight (0.90 ± 0.30 g/tail); MT body length (46.60 ± 5.50 mm), body weight (1.02 ± 0.37 g/tail). In the lead-up to the experiment, the shrimp were fed every two h with microalgae, rotifers, brine shrimp, and a compound diet consisting of 8.32% moisture, 48.65% crude protein, 5.70% crude fat, and 11.89% ash, ceasing 24 h before the experiment commenced.

### 2.3. Data Collection

Prior to initiating the formal experiment, a 96 h preliminary tolerance test was undertaken to establish the half-lethal concentration (LC50) for *L. vannamei* under conditions of high ammonia-N (130 mg·L^−1^), elevated pH (9.7), and low salinity (2 ppt). The experimental water conditions were adjusted using NH_4_CL (of analytical purity), NaOH, and oxygen-saturated fresh water, maintaining a stable temperature of 28 ± 1.5 °C and a dissolved oxygen level of at least 5 mg·L^−1^. From each family, 150 juvenile shrimp of consistent size were selected from the standard coarse pool, marked with fluorescent dye for identification, and acclimated for 24 h. Subsequently, these 150 shrimp were randomly segregated into three groups of 50 individuals each. They were allocated to three separate 7.3 m × 4.3 m × 2 m cement ponds for exposure to the specified stress conditions: high ammonia-N, high pH, and low salinity. Throughout the experiment, additional oxygen supplementation was withheld, and the occurrence of mortality was monitored at predetermined intervals. Deceased shrimp and waste materials were immediately removed, and the total mortality count was precisely documented over the course of the 96 h period.

### 2.4. Statistical Analysis

Growth and survival data were compiled and analyzed using Excel 2021. Initial analyses of the survival data for each breeding line were conducted to establish linear animal models and threshold male and female animal models for survival traits. The estimation of genetic variance components for these traits was performed using ASReml v4.2 software [15,16].

In the threshold model, the outcome category, specifically the survival status of an organism, is dictated by the value of an underlying latent variable 1, e.g., *l*_ijk_ ≤ 0, Y_ijk_ = 0 and *l*_ijk_ > 0, Y_ijk_ = 1, indicating survival. The variance of the random residuals for the latent variable l is fixed at 1. The threshold models for male and female survival traits were structured in a specific form:

Pr(Y_ijk_ = 1) = Pr(*l*_ijk_ > 0) = Φ(u + s_i_ + d_j_ + c_ij_) (1)

where Φ is the standard normal cumulative distribution function; Y_ijk_ is the survival status (death = 0, survival = 1); u is the overall mean; s_i_ is the male random effect; d_j_ is the female random effect; and c_ij_ is the full sibling family effect produced by male and female animals (i, j).

The heritability of survival was calculated using the formula [14]:

(2) $$h^{2}=\frac{4\sigma_{sd}^{2}}{2\sigma_{sd}^{2}+\sigma_{c}^{2}+\sigma_{e}^{2}}$$

where, $\sigma_{sd}^{2}$ is the additive genetic variance of males and females; $\sigma_{e}^{2}$ is the residual variance, which has a value of 1; and $\sigma_{c}^{2}$ is the full sibling family line variance.

The formula for calculating the correlation of two traits [17] is as follows:

(3) $$r=\frac{\sigma_{xy}}{\sqrt{{\sigma_{x}}^{2}{\sigma_{y}}^{2}}}$$

where, *r* is genetically or phenotypically related, $\sigma_{xy}$ denotes the additive genetic covariance or phenotypic covariance of the two traits, and ${\sigma_{x}}^{2}$ and ${\sigma_{y}}^{2}$ is their additive genetic or phenotypic variance.

The Z-score was used to test whether there was a significant difference between the heritability estimates and 0 or between the correlation estimates and 0 with the formula [18,19]:

(4) $$Z=\frac{x_{i}-x_{j}}{\sqrt{{\sigma_{i}}^{2}{\sigma_{j}}^{2}}}$$

where $x_{i}$ and $x_{j}$ is the heritability of each trait or the correlation between two traits, and ${\sigma_{i}}^{2}$ and ${\sigma_{j}}^{2}$ is the standard error of the corresponding heritability or correlation. When testing whether a genetic parameter is significantly different from 0, both $x_{i}$ and $\sigma_{j}$ are defined as 0. If |Z| ≥ 1.96, the genetic estimate is different from 0 (*p* < 0.05); if |Z| ≥ 2.58, the genetic parameter estimate is highly significantly different from 0 (*p* < 0.01).

## 3. Results

### 3.1. Descriptive Statistics of Resilience Traits in L. vannamei

**Figure 1:**
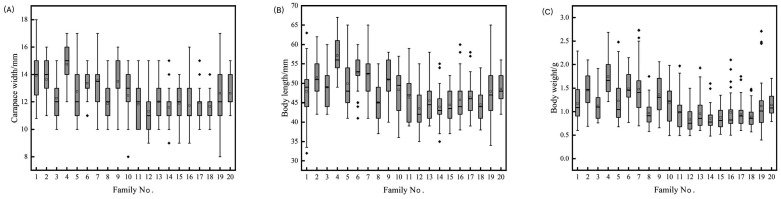
Box plot of growth-related traits of *L. vannamei* family (carapace width (**A**), body length (**B**), body weight (**C**)). Note: the maximum, minimum, and outliers are represented by “_”, “_”, and “◆”, respectively.

**Figure 2:**
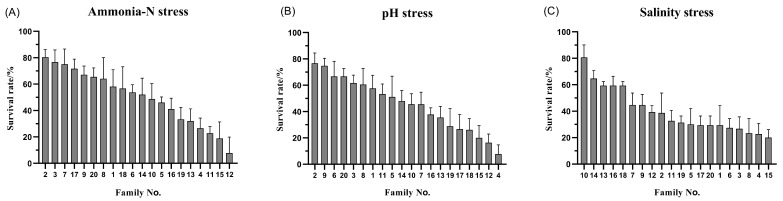
Survival of 20 families of *L. vannamei* (ammonia-N stress (**A**), pH stress (**B**), salinity stress (**C**)).

The statistics for the growth and resilience traits of *L. vannamei* are presented in Table 2. The coefficients of variation for six traits, including carapace length, ranged between 13.06% and 46.34%, with higher coefficients of variation (44.45–46.34%) for survival under stress, and 13.69%, 13.06%, and 38.46% for carapace length, body length, and body weight, respectively. These significant coefficients of variation in tolerance traits indicate substantial variability in resilience among *L. vannamei* individuals. Analysis of Figure 1 demonstrates marked variance in the median growth traits across families, with a general linear model (GLM) analysis indicating highly significant differences (*p* < 0.01) in these traits among the families. The survival rates of the various *L. vannamei* families, as illustrated in Figure 2, span from 19.52% to 92.22%, 23.33% to 92.22%, and 19.33% to 80.00% across 20 families, highlighting superior survival in families No. 2 and No. 9 under stress conditions, and lower survival rates in families No. 4 and No. 15. These descriptive statistics underscore the rich genetic diversity in the growth traits of *L. vannamei*, pointing to a significant potential for selective breeding. This diversity is crucial for ongoing research and breeding efforts, offering a basis for selecting individuals with enhanced stress tolerance for the refinement and optimization of breeding objectives.

### 3.2. Variance Components and Heritability of Resilience Traits in L. vannamei Family Lines

Table 3 details the variance components and heritability estimates for stress tolerance traits in *L. vannamei* strains. The heritability figures for high ammonia-N tolerance, high pH tolerance, and low salinity tolerance are reported as 0.44 ± 0.12, 0.41 ± 0.11, and 0.27 ± 0.08, respectively. The heritabilities of high ammonia-N and high pH tolerance were medium to high (*h*^2^ ≥ 0.30), and the heritability of low salt tolerance was medium (0.15 ≤ *h*^2^ < 0.30), which indicated that selection for stress tolerance traits with ammonia-N and pH tolerance may result in a greater genetic gain. Z-scores showed that all the heritability estimates reached significance (*p* < 0.05).

### 3.3. Analysis of Breeding Values for Stress Tolerance Traits in Various Families of L. vannamei

Table 4 identifies two family lines, No. 2 and No. 9, as exceptional performers in all three survival traits (high ammonia-N, high pH, and low salt tolerance), selected from the top 10 families based on their breeding values. These families stand out for their superior tolerance traits. Specifically, the average resistance breeding values for these two lines are 0.54, 0.67, and 0.05, respectively, marking increases of 43.63% and 86.84% and a decrease of 80.48% compared to the average breeding values of the top 10 families for each respective trait. Among the top 10 families, half exhibited identical breeding values for high ammonia-N tolerance and high pH tolerance, signifying a 50% similarity rate. Furthermore, two families shared the same breeding value for both high ammonia-N tolerance and low salt tolerance, as well as for high pH tolerance and low salt tolerance, each with a 20% occurrence rate of 20%. Two selection methods, based on phenotypic values and breeding values, were independently employed as a selection index to rank family lines of *L. vannamei*. The comparison focused on survival traits, evaluating the effectiveness of each method in determining the ranking of family lines. The top 10 family lines had identical rankings based on phenotypic values and breeding values. The family lines ranked by both methods exhibited identical breeding values of 0.37, 0.36, and 0.26 for high ammonia-N tolerance, high pH tolerance, and low salt tolerance, respectively. However, variations in selection outcomes were observed when growth traits were utilized.

### 3.4. Genetic and Phenotypic Correlations of Growth and Survival Traits in L. vannamei

Table 5 displays the phenotypic and genetic correlations between growth and stress tolerance traits. The correlations for growth traits exhibited ranges from (0.3688 ± 0.0632) to (0.6959 ± 0.0107) for phenotypic correlations and (0.3712 ± 0.2268) to (0.4321 ± 0.2165) for genetic correlations, both showing highly significant and positive correlations (*p* < 0.01) as per the Z-score test, indicating medium to high positive relationships. For the relationships between growth and stress resistance traits, phenotypic correlations varied from (0.0019 ± 0.0590) to (0.1921 ± 0.0648), with the smallest phenotypic correlation observed between high pH tolerance and body length (*r_p_* = 0.0019 ± 0.0590). Genetic correlations ranged from (0.0580 ± 0.2481) to (0.5107 ± 0.1864), with the strongest genetic correlation noted between body weight and low salt tolerance (*r_g_* = 0.5107 ± 0.1864). Comparatively, the range of genetic correlations across carapace length, body length, body weight, and tolerance to high ammonia-N, high pH, and low salt spanned from (0.0137 ± 0.2406) to (0.8327 ± 0.0781), while phenotypic correlations were observed from (0.0019 ± 0.0590) to (0.6959 ± 0.0107). These findings suggest that genetic correlations were generally stronger than phenotypic correlations in the stress resilience of *L. vannamei*.

## 4. Discussion

Quantitative traits are characteristics of organisms that vary continuously and can only be quantified through measurement, reflecting a spectrum of variability within a population [20]. These traits are notably influenced by environmental factors [21]. In aquatic organisms, such traits encompass body length, body weight, tolerance to temperature extremes, resistance to high levels of ammonia-N, and disease resistance [19]. The genetic analysis of these quantitative traits has been a focal point of recent research within the field of aquatic biology. Studies have been conducted on a variety of species, including *Crassostrea gigas* [22], *Abalone* [23], *Larimichthys crocea* [24], *Cherax quadricarinatus* [25], *Paralichthys olivaceus* [26,27,28], rainbow trout [29,30], *Atlantic salmon* [31,32,33], *Penaeus monodon* [7], *Marsupenaeus japonicus* [13], and *Portunus trituberculatus* [34]. Survival traits, which are genetically complex and influenced by multiple genes, are categorized as threshold traits within the realm of quantitative genetics. Two predominant methods, linear animal models [35,36] and threshold models [37] for males and females, have mainly been employed to gauge genetic parameters of threshold traits in aquatic animals. This study focused on estimating the genetic parameters for tolerance to high ammonia-N, high pH, and low salinity in 20 families of *L. vannamei*, revealing significant variability in stress tolerance across different families. Notably, specific families (namely No. 2 and No. 9) demonstrated superior stress tolerance across various conditions and consistently ranked among the top 10 families in resilience to all three tested stressors, distinguishing themselves markedly from other family lines.

A genetic parameter serves as a metric indicating the extent of genetic variation attributable to a heritable trait within a family line or population. This metric is deduced through the analysis of genetic relationships among individuals within a related family line or population. Commonly assessed genetic parameters include genetic variance, the genetic correlation coefficient, and heritability. In the case of *L. vannamei*, heritability estimates were calculated for traits associated with tolerance to high ammonia-N (0.44 ± 0.12), high pH (0.41 ± 0.11), and low salt (0.27 ± 0.08), utilizing the threshold model in *L. vannamei* for both genders. These values indicate heritability ranges from medium to high, highlighting the genetic basis of these stress tolerance traits. These findings are somewhat parallel to those documented by Wang [11] for stress traits involving high ammonia-N, low pH, and high salt tolerance in *Penaeus vannamei* (0.21 ± 0.06), and by Wang [9] for traits like low salt, low dissolved oxygen, and low pH tolerance in *Larimichthys crocea* (0.23, 0.10, and 0.23). Variations in heritability estimates across different studies can result from several factors, including the species being analyzed, the diversity in age and sex of the subjects, differences in growth conditions, and the use of various analytical models for evaluating the data.

The breeding value is a key metric derived from genetic parameters and trait performance data, designed to predict the future performance of offspring regarding specific traits. It is instrumental for breeders aiming to improve or enhance specific varieties in identifying individuals with superior genetic potential for parentage. Breeding values are typically calculated using methods like selection indices, and the accurate estimation of these values is crucial for crafting effective selection programs [38]. In this study, a comparison of selections based on phenotypic values and breeding values for antiretroviral survival traits resulted in identical outcomes. This agreement was noted in the selections made using both phenotypic and breeding values for the survival trait in family lines at a 10% seed retention rate. Such consistency mirrors findings in *Larimichthys crocea*, where family lines selected based on both breeding and phenotypic values for traits such as high temperature, low salt, and dry dew tolerance showed similar rates [24]. This pattern is also seen in the correlation between breeding value rankings for ammonia-N tolerance traits and phenotypic value rankings in turbot families as explored by Li [19]. However, this stands in contrast to the study by Huang [39], who utilized a population-based selection method for *Oreochromis niloticus* and observed a negative selection trend. Phenotypic value, the observable attribute of a trait in an individual, is shaped by both genetic and environmental influences. It represents the manifestation of traits under specific environmental conditions. Breeding value, conversely, signifies an individual’s genetic contribution to its progeny, devoid of environmental impact. This concept underscores the desirable genes or characteristics genetically transmitted to the next generation. The distinction between breeding value and phenotypic value selection methods is crucial for selection for various traits. The observed consistency across both breeding and phenotypic values in this study suggests a genetic underpinning for the resistance traits examined, indicating these lines may have a stable genetic basis for such traits. However, some correlation analyses between traits revealed large standard errors, likely due to the limited number of *L. vannamei* specimens used in the research [19], necessitating further examination. Nonetheless, the traits generally exhibited low to moderate positive correlations, suggesting that improvements in one trait could indirectly benefit stress tolerance, thereby supporting the selection of family lines with superior stress tolerance traits for genetic enhancement.

## 5. Conclusions

Our research uncovered pronounced disparities in stress tolerance among various *Litopenaeus vannamei* strains, specifically regarding their responses to high ammonia-N, high pH, and low salinity conditions. Notably, family lines No. 2 and No. 9 demonstrated significant resilience against elevated ammonia-N and pH levels, whereas family No. 10 was notably tolerant to reduced salinity. These particular family lines are identified as critical genetic resources for future selective breeding projects aimed at enhancing stress tolerance. The estimation of genetic parameters for a range of traits revealed moderate to high levels of heritability, accompanied by considerable additive genetic effects, highlighting the opportunity for swift genetic improvement through targeted population-based selective breeding strategies. Additionally, our findings include positive genetic and phenotypic correlations among various growth and stress tolerance traits, laying a solid theoretical foundation for the design of future selective breeding programs that prioritize stress tolerance in *L. vannamei*.

## Figures and Tables

**Table 1:** The biological data of parents.

Parent		Carapace Length (mm)	Body Length (mm)	Body Weight (g)
T	♀	5.00 ± 0.27	18.04 ± 0.63	82.58 ± 7.94
	♂	4.32 ± 0.20	16.87 ± 0.59	62.18 ± 6.46
M	♀	4.72 ± 0.27	16.44 ± 0.66	63.42 ± 7.09
	♂	4.16 ± 0.23	16.06 ± 0.79	56.34 ± 6.47

Note: T: the Dingfeng strain from Thailand; M: the Daynight Express strain from the United States.

**Table 2:** Descriptive statistics for growth and stress tolerance traits in *L. vannamei*.

Traits	Mean	SD	CV/%	Minimum	Maximum
carapace length (mm)	12.57	1.72	13.69	8.00	18.00
body length (mm)	48.09	6.28	13.06	32.00	67.00
body weight (g)	1.14	0.44	38.46	0.40	2.73
survival for ammonia-N tolerance (%)	49.94	22.20	44.45	0.00	85.71
survival for pH tolerance (%)	45.37	21.02	46.34	0.00	84.29
survival for salinity tolerance (%)	39.63	17.92	45.22	12.00	88.00

**Table 3:** Heritability and variance components of stress tolerance traits of *L. vannamei*.

Traits	Variance Components			
	$\sigma_{p}^{2}$	$\sigma_{a}^{2}$	$\sigma_{sd}^{2}$	*h* ^2^
ammonia-N tolerance (%)	4.22 ± 0.32	1.88 ± 0.63	0.47 ± 0.16	0.44 ± 0.12
pH tolerance (%)	4.14 ± 0.29	1.70 ± 0.57	0.43 ± 0.14	0.41 ± 0.11
salinity tolerance (%)	3.79 ± 0.17	1.01 ± 0.35	0.25 ± 0.87	0.27 ± 0.08

Note: $\sigma_{p}^{2}$—phenotypic variance; $\sigma_{a}^{2}$—additive genetic variance; $\sigma_{sd}^{2}$—additive genetic sire–dam variance; *h*^2^—heritability.

**Table 4:** Ranking of top 10 *L. vannamei* families based on breeding values for survival rate.

Rank	Family	Ammonia-N Tolerance -EBV	Family	pH Tolerance -EBV	Family	Salinity Tolerance -EBV
1	2	0.70	2	0.69	10	0.87
2	3	0.59	9	0.64	14	0.50
3	7	0.55	6	0.45	13	0.39
4	17	0.47	20	0.45	16	0.39
5	9	0.37	3	0.35	18	0.39
6	20	0.33	8	0.32	7	0.11
7	8	0.30	1	0.27	9	0.11
8	1	0.18	11	0.18	12	0.01
9	18	0.15	5	0.14	2	−0.01
10	6	0.09	14	0.08	11	−0.13

**Table 5:** Genetic and phenotypic correlations of growth and stress tolerance traits in *L. vannamei*.

Traits	Carapace Length	Body Length	Body Weight	Ammonia-N Tolerance	pH Tolerance	Salinity Tolerance
carapace length		0.4183 ± 0.0409	0.3712 ± 0.2268	0.3619 ± 0.2161	0.2822 ± 0.2283	0.5107 ± 0.1864
body length	0.6959 ± 0.0107		0.4321 ± 0.2165	0.2470 ± 0.2343	0.0580 ± 0.2481	0.3712 ± 0.2150
body weight	0.3734 ± 0.0611	0.3688 ± 0.0632		0.5088 ± 0.2003	0.4099 ± 0.2210	0.1485 ± 0.2514
ammonia-N tolerance	0.0852 ± 0.0609	0.0353 ± 0.0598	0.0800 ± 0.0539		0.8327 ± 0.0781	0.0220 ± 0.2403
pH tolerance	0.0624 ± 0.0604	0.0019 ± 0.0590	0.0862 ± 0.0524	0.5497 ± 0.0373		0.0137 ± 0.2406
salinity tolerance	0.1921 ± 0.0648	0.1732 ± 0.0632	0.1389 ± 0.0560	0.1350 ± 0.0653	0.1365 ± 0.0642	

Notes: phenotypic correlations are shown below the diagonal and genetic correlations are shown above the diagonal.

## Data Availability

The raw data used to support the findings of this study are available from the corresponding author upon request: F.Z., zhoufalin@aliyun.com.
